# Detection of equine herpesvirus antibodies in large‐scale donkey farms in Liaocheng area

**DOI:** 10.1002/vms3.70016

**Published:** 2024-09-13

**Authors:** Yanfei Ji, Xia Zhao, Wenqiang Liu

**Affiliations:** ^1^ College of Agricultural Science and Engineering Liaocheng University Liaocheng China; ^2^ Liaocheng Research Institute of Donkey High‐efficiency Breeding and Ecological Feeding Liaocheng China

**Keywords:** donkey, equine herpesvirus, EHV‐1, seroprevalence

## Abstract

**Background:**

Equine herpesvirus (EHV) can cause respiratory, reproductive and neurological diseases in equine animals, including donkeys. The main pathogens responsible for these diseases are EHV type 1 (EHV‐1) and EHV‐4. In this study, we collected serum samples from 230 donkeys on 27 large‐scale donkey farms to detect EHV‐1 and EHV‐4 antibodies. We analyzed the presence of EHV antibodies based on region, age and season.

**Results:**

Out of the 27 farms, 62.96% (17/27) tested positive for EHV. Of the 230 donkeys tested, 2.61% (6/230) were positive only for EHV‐1, 5.22% (12/230) were positive only for EHV‐4, and 4.78% (11/230) were positive for both EHV‐1 and EHV‐4. The highest percentage of positive donkeys (21.28%) was found in Dong'e County. The seropositivity rate among donkeys aged 1–4 years was significantly higher compared to the group of donkeys aged 0–1 year (*p* < 0.05). Additionally, the positive rate was significantly higher in fall and winter compared to spring and summer (*p* < 0.05).

**Conclusions:**

Altogether, our findings indicate that large‐scale donkey farms in the Liaocheng area have a high prevalence of EHV antibodies. Since Liaocheng is an important donkey trading market in Shandong Province, it is crucial to consider the risk of disease transmission based on our test results. This will help in early detection and prevention of EHV outbreaks.

## Graphical abstract

We found that EHV seropositivity is common in large‐scale donkey farms in Liaocheng area through EHV serological testing, and it is crucial to consider the risk of disease transmission based on our test results. This will help early detection and prevention of EHV outbreaks.

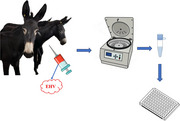

## 1 INTRODUCTION

Equine herpesvirus (EHV) infection is a contagious disease affecting equine species, characterized primarily by pneumonia and pregnancy‐related miscarriage (Abdelgawad et al., 2015). EHV is a double‐stranded DNA virus that belongs to the order Herpesvirales and family Herpesviridae. To date, nine species of equine herpesvirus have been isolated from equine hosts. These include EHV‐1, EHV‐3, EHV‐4, EHV‐6, EHV‐8 and EHV‐9, which belong to the herpesvirus subfamily Alpha equine, and EHV‐2, EHV‐5 and EHV‐7, which belong to the Gamma equine herpesvirus subfamily (Léon et al., 2008). EHV‐1 (known as equine abortion virus) and EHV‐4 (known as rhinopneumonia virus) are the most virulent, serving as the primary pathogens of EHV infections. EHV‐1 predominantly causes abortion, and neurological and respiratory diseases in pregnant mares, while EHV‐4 mainly induces respiratory illness (Lang et al., 2012).

EHV was first identified and reported in 1933 in the United States Since then, the virus has been detected in several countries and regions (Studdert et al., 1986; Turan et al., 2012). In the United States, it has been reported that approximately 80%–90% of horses have been infected with either EHV‐1 or EHV‐4 (Allen et al., 2008). EHV‐1 was found in Thoroughbred horses with respiratory disease in Hong Kong and was reported for the first time in China (Mason et al., 1989). In 2019, an outbreak of EHV‐1 infection in the Ili Kazakhstan Autonomous Region in Xinjiang, China, resulted in EHV myeloencephalopathy (EHM) and abortion, affecting 45 horses (Hu et al., 2022). Additionally, an abortion “storm” (43 out of 800) in Yili horses in Zhaosu County, northern Xinjiang, China, was ultimately confirmed to be caused by EHV‐1 infection in 2021 (Tong et al., 2022).

Donkeys, as notable members of the Equidae family, have also been reported to be infected with EHV‐1 and EHV‐4 (Harless & Pusterla, 2006). Consequently, Ali et al. (2020) isolated EHV‐1 from the foetus of an aborted female donkey in Egypt. Furthermore, Yildirim et al. (2015) conducted EHV investigations on donkeys in the northeastern region of Turkey and found high positivity rates for EHV‐1 and EHV‐4.

Although reports of EHV infection in donkeys are limited, EHV poses a significant threat to donkey health. The donkey breeding industry has recently experienced rapid development in Shandong Province, with large‐scale farming of donkeys in Liaocheng city leading the way. However, the transition from traditional breeding to large‐scale production is hindered by the issues such as abortion of pregnant female donkeys, diarrhoea in donkey foals and rhinopneumonia infection in donkeys and horses. To date, there has been no sero‐epidemiological survey of EHV in large‐scale donkey farms in China. Therefore, this study aims to determine the degree of EHV antibody positivity in large‐scale donkey farms in Liaocheng city. The study will also analyze the risk factors affecting EHV infection in order to provide reference data for the prevention and control of EHV.

## 2 MATERIALS AND METHODS

### 2.1 Sample collection

All procedures were approved by the Animal Welfare and Ethics Committee of the Institute of Animal Science, Liaocheng University (Protocol No. LC2019‐05). All methods were carried out in accordance with the relevant guidelines and regulations of the Animal Welfare and Ethics Committee of the Institute of Animal Science, Liaocheng University. Between March 2019 and February 2020, we conducted a study using 1156 Dezhou donkeys aged 0–5 years. These donkeys were held in mixed feeding across 27 officially registered large‐scale Dezhou donkey farms in Liaocheng city, as well as eight surrounding counties and cities, all located in the northern hemisphere with a temperate monsoon climate. From these donkeys, we randomly selected 230 individuals that had not received immunization against EHV. We obtained venous blood samples from these selected donkeys with the consent of their respective owners from 27 different donkey farms. Blood was collected using negative pressure blood collection tubes, and after clotting, the blood was centrifuged at 2000 rpm for 10 min to obtain serum. The serum samples were then stored frozen at −80°C for further use.

### 2.2 Detection of EHV‐1/4 antibodies

An enzyme‐linked immunosorbent assay (ELISA) was employed to detect EHV1/4 antibodies in 230 serum samples utilising the EHV1/EHV4‐Ab antibody assay kit (SVANOVIR EHV1/EHV4‐Ab; Svanova AB). The procedure was performed according to the manufacturer's instructions provided with the kit. The results were determined by measuring the optical density of the serum samples at 450 nm.

### 2.3 Data analysis

Based on the ELISA results, we investigated antibodies against EHV1 and EHV‐4 in terms of region, age, and season. SPSS 17.0 was used for the statistical analysis of the data, and 95% confidence interval were calculated. The chi‐square test (*χ*2) was used to assess significant differences in EHV‐1/4 prevalence between different regions, ages and seasons. Note that *p*‐values <0.05 are considered significant. Finally, we utilized GraphPad Prism 8 software to construct the figures.

## 3 RESULTS

### 3.1 Results of EHV antibody testing

Among the 27 donkey farms from which serum samples were collected, 62.96% (17/27) tested positive for EHV. Out of the 230 serum samples analyzed, positivity was 2.61% (6/230) for EHV‐1 only and 5.22% (12/230) for EHV‐4 only, and for both antibodies combined, it was 4.78% (11/230). The overall antibody positivity rate was 12.61% (29/230) (Table 1).

**TABLE 1:** Statistics of positive rates of EHV‐1 and EHV‐4 (where EHV is equine herpesvirus) antibodies in Liaocheng city, China.

Classify	Tested sera No.	EHV‐1 (+)	EHV‐4 (+)	EHV‐1 and 4 (+)
County				
Chiping County	43	2 (4.65%) (0.4%, 8.9%)	3 (6.98%) (1.1%, 12.9%)	1 (2.33%) (0.2%, 4.64%)
Dong'e County	47	3 (6.38%) (0.8%, 11.9%)	5 (10.64%) (3.7%, 17.6%)	2 (4.26%) (0.3%, 8.2%)
Gaotang County	28	0 (0, 5.3%)	1 (3.57%) (0.1%, 7.0%)	1 (3.57%) (0.1%, 7.0%)
Yanggu County	31	0 (0, 7.9%)	1 (3.23%) (0.2%, 6.3%)	2 (6.45%) (1.1%, 11.8%)
Dongchangfu County	28	1 (3.57%) (0.1%, 7.0%)	1 (3.57%) (0.1%, 7.0%)	1 (3.57%) (0.1%, 7.0%)
Guan County	30	0 (0, 8.8%)	1 (3.33%) (0.1%, 6.6%)	2 (6.67%) (0.7%, 12.6%)
Linqing city	11	0 (0, 20.0%)	0 (0, 20.0%)	1 (9.09%) (0.5%, 28.3%)
Shen County	12	0 (0, 18.8%)	0 (0, 18.8%)	1 (8.33%) (0.5%, 24.6%)
Age				
0 < Age ≤ 1	77	0 (0, 3.6%)	2 (2.60%) (0.2%, 5.0%)	1 (1.30%) (0, 2.6%)
1 < Age ≤ 4	85	5 (5.88%) (1.8%, 10%)	6 (7.06%) (2.7%, 11.4%)	7 (8.24%) (3.6%, 12.9%)
Age > 4	68	1 (1.47%) (0, 3.7%)	4 (5.88%) (1.6%, 10.2%)	3 (4.41%) (0.8%, 8.0%)
Season				
Spring	54	1 (1.85%) (0, 5.3%)	2 (3.70%) (0.4%, 7.0%)	1 (1.85%) (0, 5.3%)
Summer	76	0 (0, 3.75%)	5 (6.58%) (1.9%, 11.3%)	2 (2.63%) (0.3%, 5.0%)
Fall	49	1 (2.04%) (0, 5.8%)	2 (4.08%) (0.3%, 7.9%)	5 (10.20%) (3.7%, 16.7%)
Winter	51	4 (7.84%) (2.2%, 13.5%)	3 (5.88%) (0.8%, 10.9%)	3 (5.88%) (0.8%, 10.9%)
Total				
Liaocheng	230	6 (2.61%) (0.8%, 4.4%)	12 (5.22%%) (2.8%, 7.6%)	11 (4.78%) (2.5%, 7.1%)

### 3.2 Association with risk factors

**FIGURE 1:**
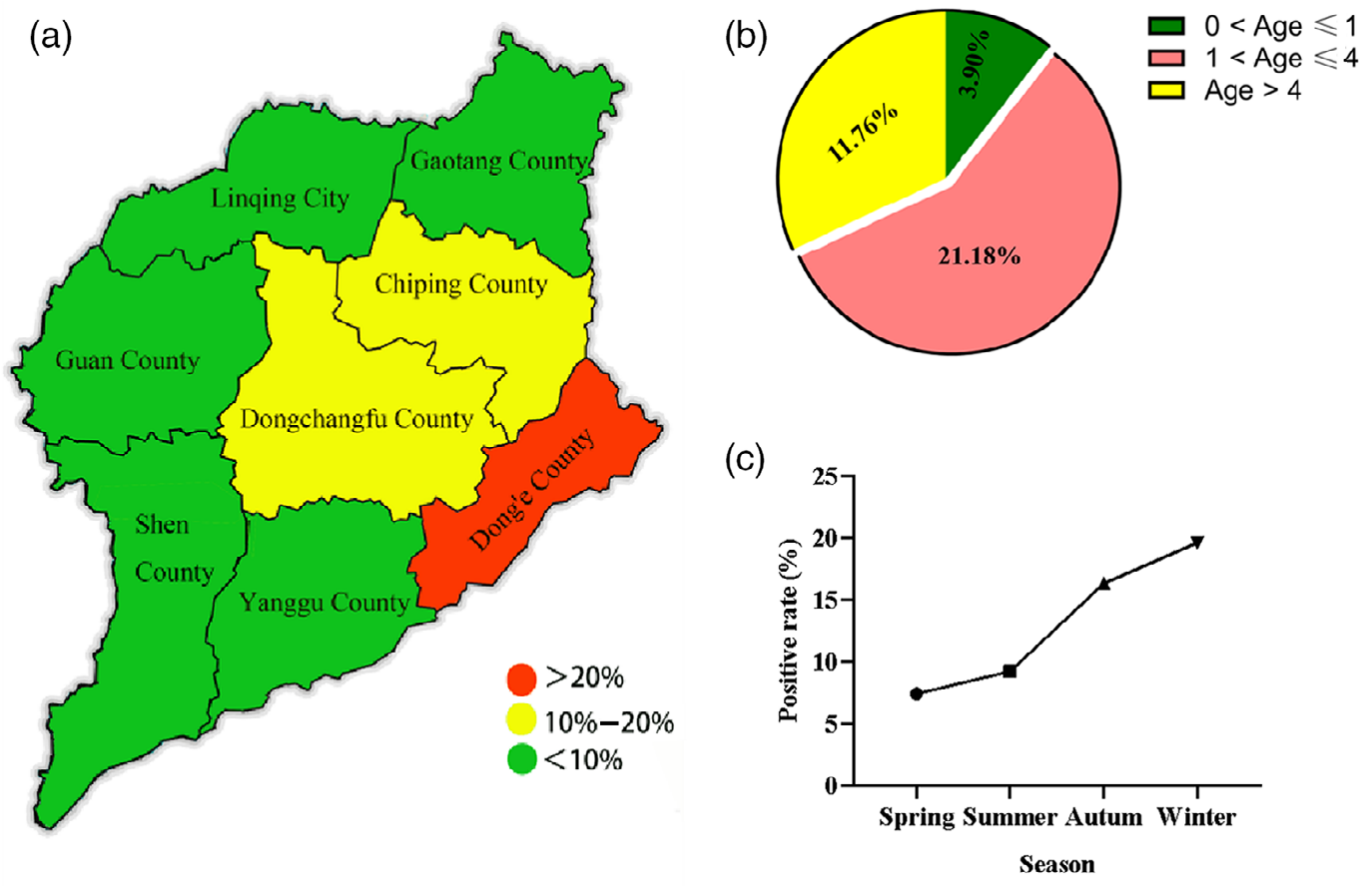
The equine herpesvirus (EHV) antibodies test results. (a) Schematic diagram of EHV antibodies positive rate in Liaocheng area. (b) Pie charts of EHV antibody positivity rate of different age groups in Liaocheng city. (c) Line graph of EHV antibody positivity rate in Liaocheng in different seasons.

Based on the simplified official map of Liaocheng area, an EHV positivity map was created using the positivity rate from each region as detailed in Table 1 (Figure 1a). The map shows that the highest EHV antibody positivity rate of 21.28% (10/47) was observed in large‐scale donkey farms in Dong'e County. In other regions, the EHV antibody positivity ranged from 8.33% to 13.95%. Table 1 and the accompanying pie chart (Figure 1b) show that donkeys aged 1–4 years had the highest antibody positivity rate, significantly higher than other age groups, with a notable difference observed in the 0−1‐year age group (*p* < 0.05). The line graph in Figure 1c depicts the seasonal serum testing results over four consecutive seasons from 2019 to 2020. The data demonstrate higher positivity rates in fall and winter compared to spring and summer. The chi‐square and p‐values for the comparison of different regions, seasons and age groups are shown in Tables 2, 3.

## 4 DISCUSSION

Large‐scale donkey farming is a significant component of the animal husbandry industry in Liaocheng city and the broader donkey industry in Shandong Province. Donkeys continue to play a crucial role in human life. Liaocheng city plays a crucial role as a transit hub for the donkey trade in Shandong Province. Thus, it is essential to focus on limiting the transmission of infectious diseases in the large‐scale donkey farms located in Liaocheng city. By doing so, we can effectively safeguard the economic stability of the donkey industry in Shandong Province. In 2021, an outbreak of EHV‐1 was reported in Spain, resulting in infections and fatalities in horses across more than 10 countries (Kubacki et al., 2021). Despite these significant outbreaks, there is a notable lack of investigation into EHV serostatus in donkeys in China. The prevalence and impact of EHV infection in donkeys remain unknown. In this study, 230 serum samples were tested, revealing that exposure to EHV infection is common in large‐scale donkey farms in the Liaocheng area. Although horses are the main hosts of EHV‐1 and EHV‐4, donkeys may be infected with EHV through contact with horses, resulting in antibody positivity (Azab et al., 2019; Cano et al., 2008; Diallo et al., 2006, 2007).

In this study, 27 large donkey farms in Liaocheng city, Shandong Province, were tested for EHV. Seventeen out of 27 donkey farms (62.98%) were antibody‐positive for EHV. As shown in Table 4, the current study found that the seroprevalence of EHV1 in donkeys in the Liaocheng area of China was 2.6% (6/230), and the seroprevalence of EHV‐4 was 5.2% (12/230). Cases of EHV1 in donkeys and horses have been reported in Turkey (Yildirim et al., 2015), Serbia (Lazić et al., 2023), Sudan (Wegdan et al., 2016), and Ethiopia (Getachew et al., 2014). Interestingly, there have also been reports of seropositive cases for EHV‐4 in donkeys in other countries, such as Turkey (Yildirim et al., 2015), Sudan (Wegdan et al., 2016), and Ethiopia (Getachew et al., 2014) (Table 4). Similar seropositive cases have been documented in other countries, including Turkey (Yildirim et al., 2015), Egypt (Azab et al., 2019), Sudan (Wegdan et al., 2016), Ethiopia (Getachew et al., 2014), and Bulgaria (Foote et al., 2003).

**TABLE 2:** *p*‐values and *χ* ^2^ for equine herpesvirus (EHV) positivity between different regions.

Region	Dong'e County	Gaotang County	Yanggu County	Dongchangfu County	Guan County	Linqing city	Shen County
Chiping County	*p* = 0.364 *χ* ^2^ = 0.842	*p* = 0.375 *χ* ^2^ = 0.787	*p* = 0.579 *χ* ^2^ = 0308	*p* = 0.688 *χ* ^2^ = 0.161	*p* = 0.613 *χ* ^2^ = 0.256	*p* = 0.613 *χ* ^2^ = 0.184	*p* = 0.605 *χ* ^2^ = 0.267
Dong'e County	N	*p* = 0.106 *χ* ^2^ = 2.608	*p* = 0.179 *χ* ^2^ = 1.809	*p* = 0.242 *χ* ^2^ = 1.366	*p* = 0.198 *χ* ^2^ = 1.659	*p* = 0.353 *χ* ^2^ = 0.861	*p* = 0.304 *χ* ^2^ = 1.056
Gaotang County	N	N	*p* = 0.727 *χ* ^2^ = 0.122	*p* = 0.639 *χ* ^2^ = 0.220	*p* = 0.698 *χ* ^2^ = 0.150	*p* = 0.837 *χ* ^2^ = 0.042	*p* = 0.896 *χ* ^2^ = 0.017
Yanggu County	N	N	N	*p* = 0.895 *χ* ^2^ = 0.017	*p* = 0.966 *χ* ^2^ = 0.002	*p* = 0.955 *χ* ^2^ = 0.003	*p* = 0.892 *χ* ^2^ = 0.019
Dongchangfu County	N	N	N	N	*p* = 0.929 *χ* ^2^ = 0.008	*p* = 0.99 *χ* ^2^ = 0.023	*p* = 0.818 *χ* ^2^ = 0.053
Guan County	N	N	N	N	N	*p* = 0.931 *χ* ^2^ = 0.008	*p* = 0.868 *χ* ^2^ = 0.028
Linqing city	N	N	N	N	N	N	*p* = 0.949 *χ* ^2^ = 0.004

**TABLE 3:** *χ* ^2^ and *p*‐values for equine herpesvirus (EHV) positivity between seasons and age groups.

Classify		*χ*2	(*χ*2) *p*‐value
Season	Spring vs. summer	4.57	0.965
	Spring vs. fall	5.71	0.159
	Spring vs. winter	6.80	0.066
	Summer vs. fall	6.83	0.092
	Summer vs. winter	5.88	0.032
	Fall vs. winter	8.82	0.669
	Spring and summer vs. fall and winter	5.13	0.023
Age	0–1 vs. 1–4	10.693	0.01
	0–1 vs. 4	3.189	0.074
	1–4 vs. 4	2.372	0.123

**TABLE 4:** Summary of seroprevalence of equine herpesvirus (EHV) (EHV1 and EHV4) in donkeys in Liaocheng, China, and other countries.

EHV	Seroprevalence in donkeys	Country	References
EHV‐1	Donkey:2.61% (6/230)	China	This study
	Donkey:33.3% (4/12)	Europe	(Léon et al., 2008)
	Donkey:51.85% (126/243)	Turkey	(Yildirim et al., 2015)
	Donkey:100% (53/53)	Serbia	(Lazić et al., 2023)
	Donkey:7.3% (6/82)	Sudan	(Wegdan et al., 2016)
	Donkey:20.2% (21/104)	Ethiopia	(Getachew et al., 2014)
EHV‐4	Donkey:5.22% (12/230)	China	This study
	Donkey:64.20% (156/243)	Turkey	(Yildirim et al., 2015)
	Donkey:58.5% (48/82)	Sudan	(Wegdan et al., 2016)
	Donkey:84.6% (88/104) Mule:100% (4/4)	Ethiopia	(Getachew et al., 2014)
EHV‐1 and EHV‐4	Donkey:4.78% (11/230)	China	This study
	Donkey:44.44% (108/243)	Turkey	(Yildirim et al., 2015)
	Donkey:43.75% (7/16)	Egypt	(Azab et al., 2019)
	Donkey:69.5% (57/82)	Sudan	(Wegdan et al., 2016)
	Donkey:74.7% (210/281)	Ethiopia	(Mekonnen et al., 2017)
	Donkey:69.7% (134/192)	Bulgaria	(Foote et al., 2003)

The rate of donkey EHV seropositivity varied in different countries and regions (Getachew et al., 2014; Lazić et al., 2023; Mekonnen et al., 2017; Wegdan et al., 2016). Our analysis revealed that Dong'e County had the highest number of positive cases of EHV, which might be attributed to the high density of donkeys in the area. Dong'e County accounts for over 50% of the total donkey population in Liaocheng. These results suggest that areas with a high density of donkeys exhibit a higher seropositivity rate, indicating a need for increased attention in these regions.

EHV antibody positivity was age‐dependent on large‐scale donkey farms in Liaocheng, with donkeys aged 1–4 years being more likely to test positive. In the present study, donkeys of all ages were at risk of seropositivity, with the highest seropositivity in adult donkeys aged 1–4 years, probably related to the disappearance of maternally derived antibodies. In addition, we found that on large‐scale donkey farms, the total number of positive serum tests collected during the fall and winter seasons was significantly higher than the total number of positive rates observed during the spring and summer seasons. Suggesting a seasonal pattern in EHV distribution among donkey herds. This seasonal variation may be related to the temperate monsoon climate of Liaocheng. During the fall and winter seasons, the decrease in temperature causes stress in donkeys, which reduces their resistance to external factors (Chenchev et al., 2011). Consistently, Foote et al. (2003) revealed that external factors further spread the EHV infection during transportation and under stress, resulting in an increased rate of animal seropositivity. Liaocheng donkey farms primarily import donkeys from the Balinzuo Banner and Arukorqin Banner of Chifeng city in Inner Mongolia, as well as Jianping County of Fuxin city in Liaoning Province. These animals are transported by land to various donkey farms in Liaocheng. Stress caused by long‐distance transport and temperature changes can easily lead to EHV infection and transmission, resulting in the production of antibodies in surrounding animals (Costantini et al., 2018; Ruiz‐Saenz et al., 2008).

## 5 CONCLUSION

In conclusion, the results of our survey indicate that antibodies against EHV‐1 and EHV‐4 are prevalent in large‐scale donkey farms in Liaocheng. Our study also analyzed various factors that could potentially affect this prevalence. These findings are crucial for guiding the development of strategies to prevent and control EHV. The high rate of EHV antibody positivity in donkey serum warrants considerable attention. It is vital for these farms to adopt measures to eliminate possible transmission routes. Additionally, strict practices such as isolation, observation, and quarantine should be enforced when introducing donkeys.

## AUTHOR CONTRIBUTIONS

**Yanfei Ji**: Conceptualization; data curation; formal analysis; visualization; writing—original draft; investigation; software. **Xia Zhao**: Conceptualization; methodology; supervision; writing—review and editing; validation; resources. **Wenqiang Liu**: Methodology; investigation; supervision; project administration; writing—review and editing; funding acquisition.

## CONFLICT OF INTEREST STATEMENT

The authors declare no conflicts of interest.

### ETHICS APPROVAL AND CONSENT TO PARTICIPATE

All procedures are approved by the Animal Welfare and Ethics Committee of the Institute of Animal Science, Liaocheng University (Protocol No. LC2019‐05). All methods are carried out in accordance with the relevant guidelines and regulations of the Animal Welfare and Ethics Committee of the Institute of Animal Science, Liaocheng University. The owners of 27 different donkey farms had agreed to collect venous blood from their donkeys.

### PEER REVIEW

The peer review history for this article is available at https://www.webofscience.com/api/gateway/wos/peer-review/10.1002/vms3.70016.

## Data Availability

The data supporting the conclusions of this case report are included in this article. All data sets can be requested from correspondence with the authors.

## References

[vms370016-bib-0001] Abdelgawad, A. A. , Hermes, R. , Damiani, A. , Lamglait, B. , Czirják, G. A. , East, M. , Aschenborn, O. , Wenker, C. , Kasem, S. , Osterrieder, N. , & Greenwood, A. D. (2015). Comprehensive serology based on a peptide ELISA to assess the prevalence of closely related equine herpesviruses in zoo and wild animals. PLoS One, 10(9), e0138370. 26378452 10.1371/journal.pone.0138370 PMC4574707

[vms370016-bib-0002] Ali, A. A. , Refat, N. A. , Algabri, N. A. , & Sobh, M. S. (2020). Fetal lesions of EHV‐1 in equine. Anais da Academia Brasileira de Ciencias, 92(suppl 2), e20180837. 32965312 10.1590/0001-3765202020180837

[vms370016-bib-0003] Allen, G. P. , Bolin, D. C. , Bryant, U. , Carter, C. N. , Giles, R. C. , Harrison, L. R. , Hong, C. B. , Jackson, C. B. , Poonacha, K. , Wharton, R. , & Williams, N. M. (2008). Prevalence of latent, neuropathogenic equine herpesvirus‐1 in the Thoroughbred broodmare population of central Kentucky. Equine Veterinary Journal, 40(2), 105–110. 18089469 10.2746/042516408X253127

[vms370016-bib-0004] Azab, W. , Bedair, S. , Abdelgawad, A. , Eschke, K. , Farag, G. K. , Abdel‐Raheim, A. , Greenwood, A. D. , Osterrieder, N. , & Ali, A. A. H. (2019). Detection of equid herpesviruses among different Arabian horse populations in Egypt. Veterinary Medicine and Science, 5(3), 361–371. 31149784 10.1002/vms3.176 PMC7155215

[vms370016-bib-0005] Cano, A. , Galosi, C. M. , Martin Ocampos, G. P. , Ramirez, G. C. , Vera, V. J. , Villamil, L. C. , & Chaparro, J. G. (2008). Equine herpesvirus 1: Characterisation of the first strain isolated in Colombia. Revue scientifique et technique (International Office of Epizootics), 27(3), 893–897. 19284057 10.20506/rst.27.3.1846

[vms370016-bib-0006] Chenchev, I. , Rusenova, N. , & Sandev, N. (2011). Seroepiemiological studies of donkeys’ blood for detection of some virus infections on ungulates. Trakia Journal of Sciences, 9, 82–86.

[vms370016-bib-0007] Costantini, D. , Seeber, P. A. , Soilemetzidou, S. E. , Azab, W. , Bohner, J. , Buuveibaatar, B. , Czirják, G. Á. , East, M. L. , Greunz, E. M. , Kaczensky, P. , Lamglait, B. , Melzheimer, J. , Uiseb, K. , Ortega, A. , Osterrieder, N. , Sandgreen, D. M. , Simon, M. , Walzer, C. , & Greenwood, A. D. (2018). Physiological costs of infection: herpesvirus replication is linked to blood oxidative stress in equids. Scientific Reports, 8(1), 10347. 29985431 10.1038/s41598-018-28688-0 PMC6037783

[vms370016-bib-0008] Diallo, I. S. , Hewitson, G. , Wright, L. , Rodwell, B. J. , & Corney, B. G. (2006). Detection of equine herpesvirus type 1 using a real‐time polymerase chain reaction. Journal of Virological Methods, 131(1), 92–98. 16137772 10.1016/j.jviromet.2005.07.010

[vms370016-bib-0009] Diallo, I. S. , Hewitson, G. , Wright, L. L. , Kelly, M. A. , Rodwell, B. J. , & Corney, B. G. (2007). Multiplex real‐time PCR for the detection and differentiation of equid herpesvirus 1 (EHV‐1) and equid herpesvirus 4 (EHV‐4). Veterinary Microbiology, 123(1–3), 93–103. 17346907 10.1016/j.vetmic.2007.02.004

[vms370016-bib-0010] Foote, C. E. , Gilkerson, J. R. , Whalley, J. M. , & Love, D. N. (2003). Seroprevalence of equine herpesvirus 1 in mares and foals on a large Hunter Valley stud farm in years pre‐ and postvaccination. Australian Veterinary Journal, 81(5), 283–288. 15084039 10.1111/j.1751-0813.2003.tb12576.x

[vms370016-bib-0011] Getachew, M. , Alemayehu, F. , Chala, C. , Amare, B. , Kassa, D. , Burden, F. , Wernery, R. , & Wernery, U. (2014). A cross‐sectional sero‐survey of some infectious diseases of working equids in Central Ethiopia. Academic Journals, 2014(9), 231–231.

[vms370016-bib-0012] Harless, W. , & Pusterla, N. (2006). Equine herpesvirus 1 and 4 respiratory disease in the horse. Clinical Techniques in Equine Practice, 5(3), 197–202.

[vms370016-bib-0013] Hu, Y. , Jia, Q. , Liu, J. , Sun, W. , Bao, Z. , Che, C. , Wu, G. , Fan, B. , Jarhen , & Ran, D. (2022). Molecular characteristics and pathogenicity of an equid alphaherpesvirus 1 strain isolated in China. Virus Genes, 58(4), 284–293. 35567668 10.1007/s11262-022-01910-y

[vms370016-bib-0014] Kubacki, J. , Lechmann, J. , Fraefel, C. , & Bachofen, C. (2021). Genome sequence of equid alphaherpesvirus 1 (EHV‐1) from a nasal swab of a Swiss horse associated with a major EHV‐1 outbreak following a show jumping event in Valencia, Spain. Microbiology Resource Announcements, 10(34), e0073221. 34435856 10.1128/MRA.00732-21 PMC8388538

[vms370016-bib-0015] Lang, A. , Vires, M. D. , Feines, S. , Müller, E. , Osterrieder, N. , & Damiani, A. M. (2012). Development of a peptide ELISA for discrimination between serological responses to equine herpesvirus type 1 and 4. Journal of Equine Veterinary Science, 32(10), S23–S23. 10.1016/j.jviromet.2013.07.044 23928223

[vms370016-bib-0016] Lazić, S. , Savić, S. , Petrović, T. , Lazić, G. , Žekić, M. , Drobnjak, D. , & Lupulović, D. (2023). Serological examinations of significant viral infections in domestic donkeys at the special nature reserve “Zasavica”, Serbia. Animals, 13(13), 2056. 37443854 10.3390/ani13132056 PMC10340027

[vms370016-bib-0017] Léon, A. , Fortier, G. , Fortier, C. , Freymuth, F. , Tapprest, J. , Leclercq, R. , & Pronost, S. (2008). Detection of equine herpesviruses in aborted foetuses by consensus PCR. Veterinary Microbiology, 126(1), 20–29. 17686590 10.1016/j.vetmic.2007.06.019

[vms370016-bib-0018] Mason, D. K. , Watkins, K. L. , & Luk, C. (1989). Haematological changes in two thoroughbred horses in training with confirmed equine herpesvirus 1 infections. The Veterinary Record, 124(19), 503–504. 2547264 10.1136/vr.124.19.503

[vms370016-bib-0019] Mekonnen, A. , Eshetu, A. , & Gizaw, D. (2017). Equine herpesvirus 1 and/or 4 in working equids: Seroprevalence and risk factors in North Showa Zone, Ethiopia. Ethiopian Veterinary Journal, 21(2), 28–39.

[vms370016-bib-0020] Ruiz‐Saenz, J. , Cruz, á. , Reyes, é. , Lopez‐Herrera, A. , & Gongora, A. (2008). Serological association between equine rinoneumonitis and equine infectious anemia viruses. Revista MVZ Córdoba, 13, 1128–1137.

[vms370016-bib-0021] Studdert, M. J. , Fitzpatrick, D. R. , Browning, G. F. , Cullinane, A. A. , & Whalley, J. M. (1986). Equine herpesvirus genomes: Heterogeneity of naturally occurring type 4 isolates and of a type 1 isolate after heterologous cell passage. Archives of Virology, 91(3–4), 375–381. 3022687 10.1007/BF01314296

[vms370016-bib-0022] Tong, P. , Duan, R. , Palidan, N. , Deng, H. , Duan, L. , Ren, M. , Song, X. , Jia, C. , Tian, S. , Yang, E. , Kuang, L. , & Xie, J. (2022). Outbreak of neuropathogenic equid herpesvirus 1 causing abortions in Yili horses of Zhaosu, North Xinjiang, China. BMC Veterinary Research, 18(1), 83. 35232435 10.1186/s12917-022-03171-1 PMC8886757

[vms370016-bib-0023] Turan, N. , Yildirim, F. , Altan, E. , Sennazli, G. , Gurel, A. , Diallo, I. , & Yilmaz, H. (2012). Molecular and pathological investigations of EHV‐1 and EHV‐4 infections in horses in Turkey. Research in Veterinary Science, 93(3), 1504–1507. 22401978 10.1016/j.rvsc.2012.01.019

[vms370016-bib-0024] Wegdan, H. A. , Intisar, K. S. , Shaza, M. M. , Algezoli, O. A. , Ballal, A. , Ihsan, H. A. , Sahar, M. E. , Baraa, A. M. , Manal, H. S. , Muna, E. A. , Taha, K. M. , Nada, E. M. , & Ali, Y. H. (2016). Serological detection of equine herpes virus (EHV) type 1 and 4 in Sudan. British Microbiology Research Journal, 14(6), 1–6.

[vms370016-bib-0025] Yildirim, Y. , Yilmaz, V. , & Kirmizigul, A. (2015). Equine herpes virus type 1 (EHV‐1) and 4 (EHV‐4) infections in horses and donkeys in northeastern Turkey. Iranian Journal of Veterinary Research, 16(4), 341–344. 27175200 PMC4782672

